# Phenotype and Functions of Natural Killer Cells in Critically-Ill Septic Patients

**DOI:** 10.1371/journal.pone.0050446

**Published:** 2012-12-06

**Authors:** Jean-Marie Forel, Laurent Chiche, Guillemette Thomas, Julien Mancini, Catherine Farnarier, Céline Cognet, Christophe Guervilly, Aurélie Daumas, Frédéric Vély, François Xéridat, Eric Vivier, Laurent Papazian

**Affiliations:** 1 Faculté de médecine, Aix-Marseille Université, URMITE UMR CNRS 7278, Marseille, France; 2 APHM, Hôpital Nord, Réanimation des détresses Respiratoires et des Infections sévères, Marseille, France; 3 Centre d’Immunologie de Marseille-Luminy (CIML), Aix Marseille Université, INSERM U, CNRS UMR7280, Marseille, France; 4 Laboratoire d’Immunologie, Assistance Publique des Hôpitaux de Marseille, Hôpital de la Conception, Marseille, France; 5 APHM, Service de Santé Publique, Hôpital de la Timone, Marseille, France; 6 Aix-Marseille Univ, UMR912, Inserm, IRD, Marseille, France; Hannover Medical University (MHH), Germany

## Abstract

**Rationale:**

Natural killer cells, as a major source of interferon-γ, contribute to the amplification of the inflammatory response as well as to mortality during severe sepsis in animal models.

**Objective:**

We studied the phenotype and functions of circulating NK cells in critically-ill septic patients.

**Methods:**

Blood samples were taken <48 hours after admission from 42 ICU patients with severe sepsis (*n* = 15) or septic shock (*n* = 14) (Sepsis group), non-septic SIRS (*n* = 13) (SIRS group), as well as 21 healthy controls. The immuno-phenotype and functions of NK cells were studied by flow cytometry.

**Results:**

The absolute number of peripheral blood CD3–CD56^+^ NK cells was similarly reduced in all groups of ICU patients, but with a normal percentage of NK cells. When NK cell cytotoxicity was evaluated with degranulation assays (CD107 expression), no difference was observed between Sepsis patients and healthy controls. Under antibody-dependent cell cytotoxicity (ADCC) conditions, SIRS patients exhibited increased CD107 surface expression on NK cells (62.9[61.3–70]%) compared to healthy controls (43.5[32.1–53.1]%) or Sepsis patients (49.2[37.3–62.9]%) (p = 0.002). Compared to healthy (10.2[6.3–13.1]%), reduced interferon-γ production by NK cells (K562 stimulation) was observed in Sepsis group (6.2[2.2–9.9]%, p<0.01), and especially in patients with septic shock. Conversely, SIRS patients exhibited increased interferon-γ production (42.9[30.1–54.7]%) compared to Sepsis patients (18.4[11.7–35.7]%, p<0.01) or healthy controls (26.8[19.3–44.9]%, p = 0.09) in ADCC condition.

**Conclusions:**

Extensive monitoring of the NK-cell phenotype and function in critically-ill septic patients revealed early decreased NK-cell function with impaired interferon-γ production. These results may aid future NK-based immuno-interventions.

**Trial Registration:**

NTC00699868.

## Introduction

Critically ill patients are admitted in intensive care units (ICU) following various conditions such as sepsis, trauma, pancreatitis, hemorrhagic shock, or surgery. All these conditions share common host-response characteristics, referred to as systemic inflammatory response syndrome (SIRS) [1]. Both sepsis and non-sepsis-related SIRS are characterized by an exacerbated inflammatory response [2], and mortality remains high, especially in the settings of severe sepsis and septic shock [3]. A common feature of these patients resides in alteration of their immune status, termed as compensatory anti-inflammatory response syndrome (CARS), which is thought to render them more susceptible to nosocomial infections [4], and to lead to increased morbidity and mortality in the ICU [5]. To date, various immunotherapies have failed to prevent the consequences of SIRS/CARS in severely septic patients, and efforts are still needed to fully understand the effects of the inflammatory and anti-inflammatory processes on the immune status of these patients [2], [6].

Although monocytes from patients with SIRS or sepsis have been studied [7], [8], NK cells have received much less analysis. NK cells, found within the bloodstream, are also abundant in some tissues such as the lung [9], an organ particularly prone to dysfunction in ICU patients [10]. Importantly, murine experiments have shown collectively a deleterious proinflammatory effect of NK cells [11]–[16]. In these models, NK cells were a major source of interferon (IFN)-γ, a potent immuno-stimulatory cytokine [17], and early depletion of NK cells led to clear improvements in survival of sepsis-challenged mice [11]–[16]. Thus, one might expect NK cells to contribute to the amplification of the inflammatory response during the early steps of severe sepsis in humans too. The identification of over-activated NK cells during the early phase of severe sepsis and septic shock in critically-ill patients, mirroring what has been observed in animal models, could provide a unique opportunity to define NK cell-based immunotherapeutic interventions. However, available human data are scarce. Most studies are limited to quantitative assessment of NK cells [18]–[22] (Table S1). Studies have addressed NK-cell functionality in patients with septic shock, but have been limited to cytotoxic functions [23]–[25] and used samples obtained 7 days after ICU admission [25] or have included immunocompromised (*i.e.*, cancer) patients [23].

Herein, we aimed to quantitatively and qualitatively characterize at ICU admission circulating NK cells of critically-ill septic patients.

## Methods

### Study Design

This prospective cohort study was conducted in the medical ICU of Assistance Publique - Hôpitaux de Marseille University Hospital (France). The study was approved by the Sud-Méditerranée V Ethics Committee and written informed consent was obtained from all patients or, according to French law, from their proxies when patients were not able to understand. The study, which one goal was to evaluate NK cell status before cytomegalovirus reactivation during the ICU stay (Methods S1), included a factorial study that is presented herein. The principal aim was the quantitative and qualitative monitoring of NK cells status in the early phase of critically-ill septic patients in comparison to healthy controls as well as patients with severe non-septic SIRS. Secondary aims included comparison between severe sepsis and septic shock. Potential explanatory factors for observed modifications (circulating cytokines levels, NK activating/inhibiting receptors surface expression) were also investigated as an exploratory part of this study.

During a 2-year period, all consecutive patients meeting inclusion criteria were eligible. Inclusion criteria included being aged >18 years and the absence of any immunodeficiency prior to ICU admission (Methods S1). All enrolled patients had blood samples drawn within the first 48 h of ICU admission. Lymphocyte subset counts (CD3^+^, CD4^+^, CD8^+^, CD19^+^,CD56^+^, CD16^+^, HLA-DR^+^) were first performed using flow cytometry on fresh whole-blood samples. Then, peripheral blood mononuclear cells (PBMCs) were isolated by Ficoll–Hypaque density gradient centrifugation (Eurobio, Courtaboeuf, France), counted, and stored in liquid nitrogen vapor. Serum was frozen at –80°C.

The constitution of patients groups was done as follows. First, to avoid any influence of CMV status on NK-cell phenotype, we selected from the whole cohort patients with CMV seropositivity at admission [26]. Then, we constituted different groups: those with sepsis (referred to thereafter as “Sepsis group”), including those with septic shock and those with severe sepsis, and those with SIRS of non-infectious origin (referred to thereafter as “SIRS group”) (Methods S1). Immunological analyses were then performed for these patients (n = 42) on frozen samples. Range values defining NK cell subsets and functions in unmatched healthy controls (n = 21; age range 25–60 years) were used to define “normal” values. They were analyzed in the same technical as for ICU patients to avoid technical bias.

### Immunological Analyses

#### Immuno-phenotype of NK cells

NK cells were defined as CD3–CD56^+^ cells within the lymphocyte gate, and the various monoclonal antibodies (mAbs) were used to define human subsets of NK cells (Methods S1).

#### NK-cell effector functions

NK-cell effector functions were tested in a single-cell assay using CD107 (LAMP) mobilization and IFN-γ production, as previously described [27] (Methods S1). To directly assess NK-cell function, a flow cytometric cytotoxicity assay based on staining with carboxyfluorescein diacetate succinimidyl ester (CFSE) was used (Methods S1).

### Serum Cytokines

Levels of various cytokines in serum were determined. The immunoassays were performed following the manufacturer’s instructions (Methods S1).

### Statistical Analyses

Comparisons between healthy, SIRS and Sepsis groups were carried out using the non-parametric Kruskal–Wallis test for unpaired continuous data, and Pearson Chi-square test for categorical variables. Then, pairwise comparisons between 3 groups (healthy, SIRS, Sepsis) were carried out using the Kruskal-Wallis post–hoc methods for multiple comparisons adjusted by step-up Simes method [28] (Methods S1). The Mann-Whitney U test was used when two groups were just compared. Correlations were assessed by the Spearman correlation test. Data were expressed as median [IQR] or as counts (%), as required. A *p*-value (two-tailed) threshold of 0.05 was considered statistically significant.

## Results

### Demographic and Clinical Characteristics of the Study Population

From the patients enrolled during the study period, 42 who corresponded to the predefined criteria were selected to constitute the groups: 29 patients in the Sepsis group (including 15 with septic shock and 14 with severe sepsis) and 13 patients in the SIRS group. The times between ICU admission and sampling were similar between all groups (Table 1). Sepsis and SIRS groups were comparable for characteristics on admission. As expected, the severity on admission as well as the proportion of patients receiving mechanical ventilation or meeting ARDS criteria were significantly increased in patients with septic shock compared to those with severe sepsis (Table 1). Groups also showed differences concerning outcomes, with a trend towards higher morbidity and mortality in the Sepsis group compared with the SIRS group (Table 1).

**Table 1:** Characteristics on admission and outcome of ICU patients.

				Sepsis group (*n* = 29)		
	SIRS group*n* = 13	Sepsis group*n* = 29	*p*	Severe sepsis*n* = 15	Septic shock*n* = 14	p*
**Characteristic on admission to ICU**						
Age (years), median [IQR]	66 [63–71]	64 [57–73]	ns	61 [55–73]	67 [63–75]	0.057
Gender: Male no. (%)	6 (46)	17 (59)	ns	8 (53)	9 (64)	ns
Time of sampling from ICU admission (days), median[IQR]	1 [1]–[2]	1 [1]–[2]	ns	1 [1]–[2]	1 [1]–[2]	ns
SAPS II score, median [IQR]	43 [34–61]	49 [40–58]	ns	43 [26–57]	52 (47–73]	0.051
SOFA score, median [IQR]	7 [6]–[11]	9 [7]–[11]	ns	8 [4]–[9]	10 [8]–[11]	0.039
Vasopressors, no. (%)	5 (38.5)	14 (48.3)	ns	0 (0)	14 (100)	<0.001
Mechanical ventilation (MV), no. (%)	10 (77)	25 (86)	ns	11 (73.3)	14 (100)	0.037
ARDS, no. (%)	1 (7.7)	7 (25)	ns	0 (0)	7 (50)	0.002
**Outcome during ICU stay**						
Mortality, no. (%)	1 (7.7)	6 (20.7)	ns	3 (20)	3 (21.4)	ns
Nosocomial bacterial infection, no. (%)	7 (53.8)	11 (37.9)	ns	8 (53.3)	3 (21.4)	0.08
Ventilator–associated pneumonia, no. (%)	7 (53.8)	7 (24.1)	0.06	6 (40)	1 (7.1)	0.039
CMV reactivation, no. (%)	2 (15.4)	12 (43)	0.08	6 (40)	6 (42.9)	ns
ICU length of stay (days), median [IQR]	14 [8]–[17]	20 [10]–[34]	ns	25 [10–49]	18 [8]–[24]	ns
Length of MV (days), median [IQR]	8 [6]–[10]	16 [5]–[32]	ns	19 [5]–[47]	14 [5]–[24]	ns

Sepsis group includes patients with severe sepsis and septic shock. CMV: cytomegalovirus; ICU: intensive care unit; MV: mechanical ventilation; SIRS: systemic inflammatory response syndrome; SOFA: sepsis-related organ-failure assessment.

*p:* Comparison between SIRS and Sepsis groups using Mann-Whitney U test or Pearson Chi-Square test. p*: Comparison between severe sepsis and septic shock using Mann-Whitney U test or Pearson Chi-Square test.

### Numbers of NK Cells in ICU Patients

The proportions of CD3–CD56^+^ NK cells (% of total lymphocytes) were similar in Sepsis group patients, SIRS group patients and healthy controls (Table S2), with no difference between patients with severe sepsis and septic shock. The absolute number of peripheral blood CD3–CD56^+^ NK cells was reduced similarly in Sepsis and SIRS groups (69/mm^3^ [55–106] and 111/mm^3^ [57–142] respectively) compared to healthy controls (203/mm^3^ [149–284], *p*<0.001). No difference was observed between patients with severe sepsis (68/mm^3^ [64–82]) and septic shock (75/mm^3^ [30–137]). NK-cell lymphopenia was part of the severe and global lymphopenia that occurred in ICU patients, and involved B cells (CD19^+^) and T cells (CD3^+^CD4^+^ and CD3^+^CD8^+^). There was no difference observed in absolute and relative counts of different T-cell subsets between Sepsis and SIRS groups, but more severe B-cell lymphopenia (CD19^+^) was observed in the Sepsis group (Table S2).

### NK-cell Effector Functions in ICU Patients

**Figure 1:**
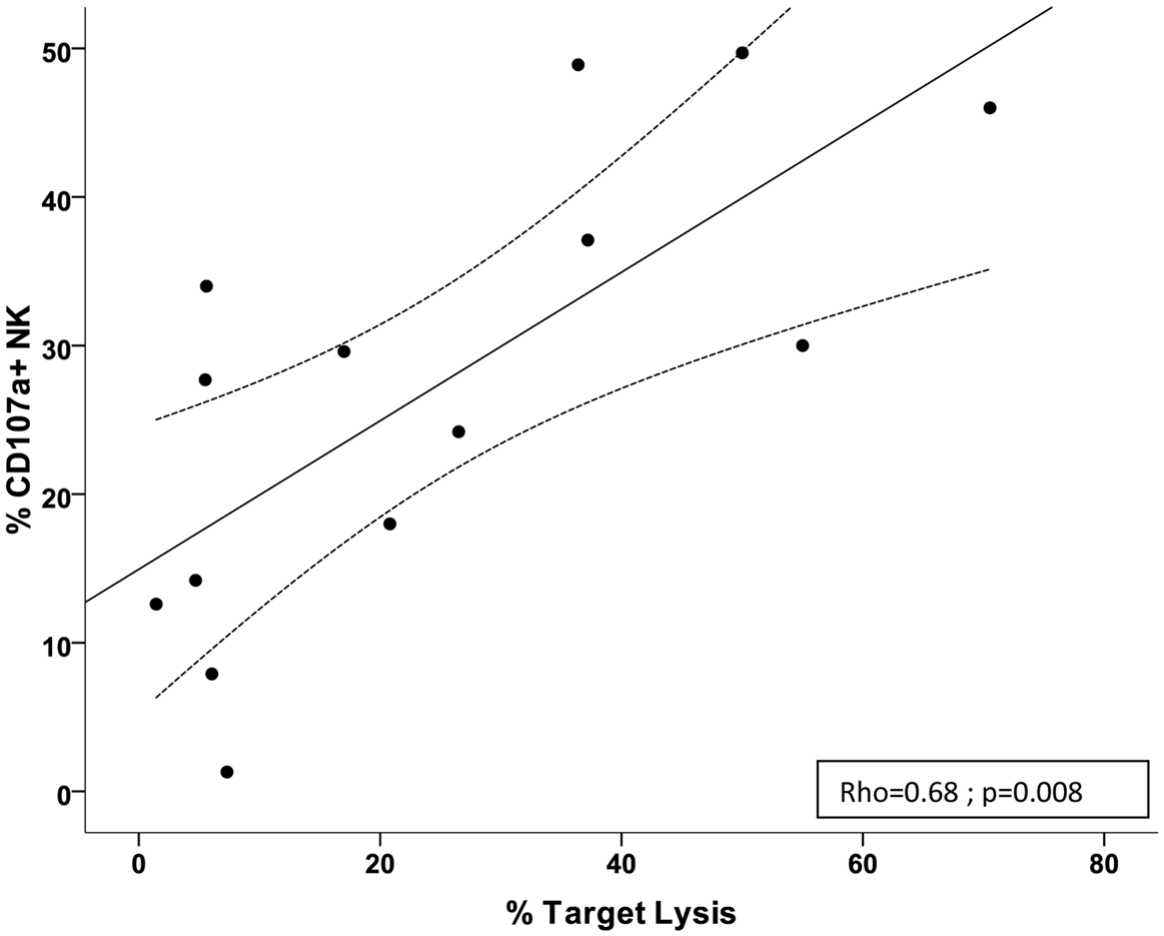
Evaluation of cytotoxic functions of NK cells in ICU patients. Correlation between the direct cytotoxicity CFSE-based assay and the degranulation CD107a expression assay to evaluate cytotoxic functions of NK cells in ICU patients (*n* = 14). Results are expressed as % lysis of target cell for the CFSE-assay, and as % NK-cell expressing CD107a for the degranulation assay. Effector–target ratio is 50/1 (PBMC/K562) for the CFSE-assay, and 2.5/1 (NK/K562) for the CD107a expression assay.

Because of the severe lymphopenia observed in the ICU patients, we were only able to evaluate the cytotoxic function (direct killing) of NK cells using a direct cytotoxicity CFSE-based assay for 14 of the 42 patients. We observed no significant differences between groups (data not shown) but, due to the low number of patients in each group, this analysis was poorly interpretable. Nevertheless, for each ICU patient that could be evaluated for direct cytotoxicity (CFSE-based assay) and for degranulation (LAMP or CD107-based assay) of NK cells, both results were strongly correlated (Rho = 0.68, p = 0.008) (Figure 1). This confirms that CD107 expression is a valuable tool to indirectly assess NK cytotoxic function in the setting of ICUs.

**Figure 2:**
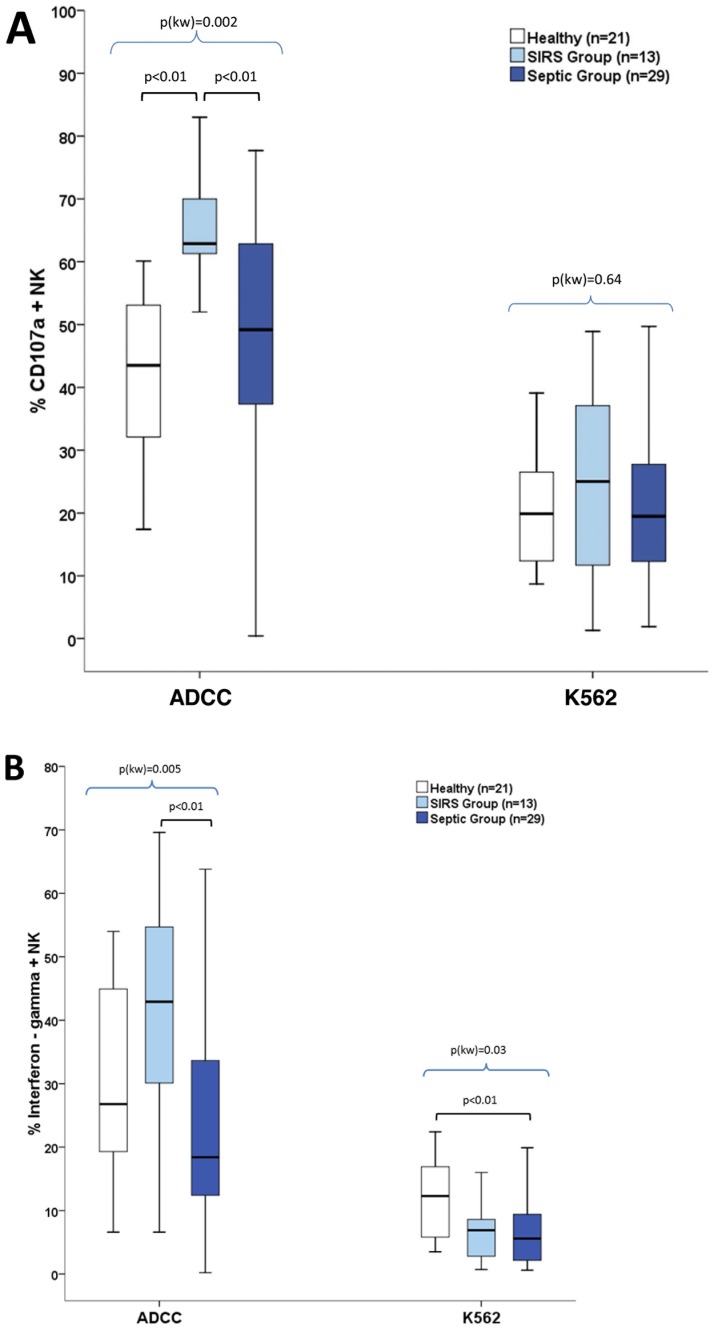
Evaluation of NK cell functions in ICU septic patients. NK degranulation (**A**) and intracellular production of IFN-γ (**B**) of ICU patients with Sepsis, SIRS, and healthy controls. **A:** Degranulation responses by CD107a cell-surface expression (% of positive NK cells) against K562 target cells (natural cytotoxicity) or P815 mouse mastocytoma cells coated with rabbit anti-mouse lymphocyte antibodies (ADCC). **B:** Intracellular IFN-γ expression (percentage of positive NK cells), against K562 target cells or P815 (ADCC). Number of samples from each group: Sepsis group (*n* = 29), SIRS group (*n* = 13), and healthy controls (*n* = 21). A black bar inside the box-and-whiskers plots indicates the median. *p(kw)*: Comparison between healthy, SIRS and Sepsis groups by Kruskal-Wallis test. *p*: pairwise comparisons between groups (healthy, SIRS, Sepsis) by Kruskal-Wallis post–hoc methods for multiple comparisons adjusted by step-up Simes method.

NK-cell functions were further investigated using *in vitro* degranulation (CD107-based assay) and cytokine-secretion assays. We first tested the cell-surface induction of CD107a (LAMP1) in all patients, which reflects NK-cell degranulation capacity when triggered by the prototypical K562 tumor cell line or antibody-coated target cells (referred to as antibody-dependent cell cytotoxicity [ADCC] conditions thereafter) (Figure 2A). Under natural cytotoxic conditions (with K562 target cells), no difference in CD107 expression was observed between Sepsis group (21 [12]–[28] %), SIRS group (25 [12]–[37] %) and healthy controls (17 [12]–[22] %, *p* = 0.64) (Figure 2A). Under ADCC conditions, no difference in CD107 expression was observed between Sepsis group patients (49.2 [37.3–62.9] %) and healthy controls (43.5 [32.1–53.1] %) as well as between patients with severe sepsis (49.8 [42.8–64.5] %) and septic shock (39.7 [33.8–54.6] %). Conversely, SIRS group patients exhibited increased CD107 surface expression on NK cells (62.9 [61.3–70] %) compared to healthy controls (43.5 [32.1–53.1] %, p<0.01) as well as compared to Sepsis group patients (49.2 [37.3–62.9] %, p = <0.01) (Figure 2A) suggesting increased cytotoxicity/degranulation.

We then explored IFN-γ secretion by NK cells under the same conditions of stimulation (Figure 2B). Under stimulation with K562 cells a significantly reduced IFN-γ production was observed only in Sepsis group patients (6.2 [2.2–9.9] %) compared to healthy controls (10.2 [6.3–13.1] %, p<0.01), especially in those with septic shock (3.0 [1.9–10.7] %). Under ADCC conditions, a trend toward decreased IFN-γproduction was also observed in Sepsis group patients (18.4 [11.7–35.7] %) compared to healthy controls (26.8 [19.3–44.9] %, *p* = 0.09), whereas SIRS group patients exhibited a trend to increased IFN-γ production (42.9 [30.1–54.7] %) compared to healthy controls (p = 0.09). Moreover, the SIRS group patients exhibited increased IFN-γ production (42.9 [30.1–54.7] %) compared to Sepsis group patients (18.4 [11.7–35.7] %, p<0.01). Collectively, these analyses revealed an unexpected “normal” (instead of over-activated) NK-cell functional status concerning cytotoxic/degranulation capacities, and even decreased IFN-γ production capacities in critically ill septic patients. Conversely, ICU patients from SIRS group exhibited an over-activated status that involved both IFN-γ production and cytotoxic functions. We then performed further analyses to look for potential mechanisms underlying these results.

### Phenotype of NK Cells in ICU Patients

Circulating NK cells were phenotyped to define subsets of NK cells according to surface markers and to evaluate expression of activating and inhibitory receptors susceptible to being influenced by NK-cell function. The relative proportions CD3–CD56^dim^ and CD3–CD56^bright^ NK-cell subsets were similar in patients with Sepsis, SIRS (Table 2) and comparable to the normal values of our laboratory. CD56^dim^ NK cells were the main source of IFN-γ secretion (data not shown). Except for higher proportion of KIR3DL1^+^ NK cells, no difference in expression of either activating (i.e., CD16, NKp30, NKp46, and NKG2D) or inhibitory membrane receptors (i.e., KIR, NKG2A) was observed between Sepsis group and SIRS group patients (Table 2), as well as between patients with severe sepsis or septic shock (data not shown).

**Table 2:** NK-cell phenotype of ICU patients with SIRS and Sepsis.

	SIRS group*n* = 13	Sepsis group*n* = 29	*p*
CD56 ^dim^, %	94.6 [90.9–96]	95.0 [91.9–96.2]	ns
CD56 ^bright^, %	5.4 [4–9.1]	5.0 [3.8–8.1]	ns
CD94, %	63.4 [53.1–68.7]	47.5 [41.8–63.4]	ns
NKp30, MFI	182 [145–338]	215 [123–289]	ns
NKp46, MFI	638 [462–1137]	578 [399–937]	ns
CD16, %	94.3 [87–96.3]	94.4 [81.1–96.9]	ns
NKG2A, %	53.9 [38.7–59.6]	47.2 [33.3–57.1]	ns
NKG2C, %	13.4 [11.3–18.3]	10.2 [8.3–18.1]	0.071
NKG2D, MFI	520 [350–591]	450 [374–506]	ns
CD25, %	9.3 [6.4–12.7]	6.1 [4.1–9.7]	0.056
CD158a (KIR 2DL1), %	16.3 [10.5–18.9]	14.3 [8.8–18.5]	ns
CD158 a,h (KIR 2DL1–2DS1), %	0 [0–8]	0 [0–4]	ns
CD158h (KIR 2DS1), %	0 [0–6]	2 [0–7]	ns
CD158e (KIR3DL1), %	6 [0–14]	18 [10]–[32]	0.037

Results are expressed as median percentage [IQR] of positive NK cells for activating or inhibitory receptors, except for NKG2D, NKp30, and NKp46, which are expressed as median MFI. *p:* Comparison between SIRS and Sepsis groups using Mann-Whitney U test.

### Serum Cytokine Levels in ICU Patients

We then tested whether NK-cell functions could be associated with changes in circulating cytokines. Except for higher IL-1β concentrations, there were no significant differences in the concentrations of circulating TNF-α, IFN-γ, IL-6, IL-10, IL-12, IL-15, IL-18, TGF-β1, and TGF-β2 between Sepsis and SIRS groups (Table S3). Interestingly, patients with septic shock exhibited lower concentrations of two major NK-cell stimulating cytokines, IL-12 (*p* = 0.035) and IL-18 (*p* = 0.054) than those with severe sepsis (Table S3).

### NK Status on Admission and Outcomes

Considering ICU morbidity, NK-cell functions at admission to the ICU were not correlated to the further occurrence of nosocomial infections (including bacterial VAP and CMV reactivation) or to the length of mechanical ventilation or ICU stay. NK-cell functions at admission to the ICU were also not correlated with severity scores (data not shown). Considering mortality in all ICU patients, T-cell lymphopenia was more severe among non-survivors (654/mm^3^ [504–1030] in survivors vs. 392/mm^3^ [313–407] in non-survivors, *p* = 0.047). But no quantitative (NK-cell count and percentage) or qualitative (NK-cell phenotype and functions) differences were observed between survivors and non-survivors, either in overall ICU patients or in the subset of septic patients (data not shown).

## Discussion

Mortality from severe sepsis and septic shock is still dramatically high, in spite of major advances in critical-care medicine [3]. Massive activation of mononuclear phagocytes by bacterial components and release of proinflammatory cytokines [2] are rapidly compensated for by an anti-inflammatory response, also called immunoparalysis or CARS, which can cause secondary nosocomial infections and death [4], [5]. Patients may exhibit both hyper-inflammation and immune paralysis concomitantly, with one being transiently dominant over the other [29]: this may partly explain the failure of anti-inflammatory drugs in these conditions. Thus, only better characterization of the nature of immune responses during severe sepsis and septic shock can enable design of appropriate immuno-interventions. In humans, most previous studies have reported deactivation of monocytes [7], [30], or the massive apoptosis of T and B lymphocytes [21], [31], whereas NK cells have been understudied. However, as part of the innate immune system, NK cells participate in the early response to microbial infections, involving both IFN-γ secretion and perforin-dependent target-cell elimination [32]. NK cells may also shape the adaptive immune response through cytokine release or direct interaction with dendritic cells [33]. Indeed, various murine models of severe sepsis, which show that NK-cell depletion improves survival, support detrimental over-activation of NK cells involved in the amplification of the inflammatory response [16]. In particular, NK cells are believed to be a key source of pro-inflammatory IFN-γ during the early stages of severe sepsis [17].

Herein, we report, to the best of our knowledge, the first study to provide extensive assessment of circulating NK cells in critically ill septic patients. Unexpectedly, and in apparent contradiction to murine data, we only observed an over-activated status in SIRS patients, but not in Sepsis patients, with the latter even exhibiting decreased production of IFN-•γ.especially in those who presented with septic shock. Concerning IFN-γ production by NK cells, no previous evaluation has been performed in the setting of ICUs. Conversely, previous studies have addressed the cytotoxic functions of NK cells in various inflammatory conditions, including sepsis [23]–[25], but also other causes of non-septic SIRS (i.e., major surgery or severe burns) [34]–[38]. However, comparisons between our results and previous studies should be cautious because of the different settings (patients’ severity and/or comorbidities and timing of sampling) and the methods used to assess NK cytotoxic functions (Table S1). First, most previous studies have used samples taken at a later time. For example, in the study by Muller et al., analyses were performed on patients with septic shock, but only at 7 days after admission to the ICU [25]. The relatively early sampling time in our study might explain why we did not observe decreased NK cytotoxic functions in our septic-shock patients. Conversely, the difference between our non-septic SIRS patients with overall “activated” status and the “deactivated” status in patients with burns (reported by others) may seem more difficult to explain. However, it is important to take into account that we observed over-activation only in ADCC conditions, and not in K562 conditions, which was the only condition tested in previous studies. This point underlines the importance of completely evaluating the functions of specific cells (ie, NK cells) with various stimuli, to address the different activation pathways of these cells. Second, most studies performed to date have only evaluated the absolute and/or relative number of circulating NK cells in severe septic or non-septic conditions (Table S1). We observed a normal percent of NK cells in all groups of ICU patients, which is in complete agreement with most other studies. Interestingly, Muller et al. and Andaluz et al. [25], [18] found increased percentages of NK cells in septic patients, and the mortality of their patients was twice that of the study of Venet et al. [20] and our study, which reported normal percentages of NK cells. We observed that NK lymphopenia was reported in most previous studies and it is hypothesized that NK cells migrate from the blood to inflamed tissue [31]. This NK lymphopenia was part of a global lymphopenia that involved B and T lymphocytes, which was more severe among septic patients and among non-survivors within the overall ICU population, as has been already reported [19], [31]. Conversely, and in agreement with the results of de Pablo et al. [39], we could not observe any correlation between the numbers of circulating NK cells on admission and ICU mortality (even in the septic patients), as recently reported in a recent study [18]. We could not correlate NK-cell count or function on admission to the further occurrence of nosocomial infection. However, the small sample size of this pilot study precludes any firm conclusions on these two points.

Additional to the global characterization of NK cells, we performed an extended phenotypic analysis with the hypothesis that disequilibrium between inhibitory and/or activating surface receptors might explain differences in NK function between SIRS (over-activated) and Sepsis group patients (normal or decreased functions). Indeed, potent NK-cell effector functions, such as cytotoxicity and cytokine production, require dynamic integration of signals derived from multiple receptors. There are numerous activating NK-cell receptors that belong to different receptor families and contain various cytoplasmic signaling domains [40]. Interestingly, the only significant difference observed was a higher proportion of NK cells expressing the inhibitory receptor KIR3DL1 in patients with Sepsis compared to non-septic SIRS. Finally, we also explored the possible role of circulating cytokines to explain NK functional differences between ICU patients, especially with regards to decreased IFN-γ production by NK cells. Monocyte-derived proinflammatory cytokines, such as IL-12, IL-15, and IL-18 (especially in combination), positively regulate IFNγ- secretion by NK cells [41], [42], whereas IL-10 and TGF-β can act as negative regulators of NK-cell IFNγ- production, which leads to a state endotoxin tolerance [43], [44]. Interestingly, among septic patients, those with septic shock that exhibited the most important reduction concerning IFN-γ production showed no difference in levels of IL-10 or TGF-β, but lower levels of IL-12 and IL-18 than those with severe sepsis. The possible role of increased expression of inhibitory NK receptors and/or decreased NK-cell stimulating cytokines warrants further validation.

This study has some limitations. First, evaluation of direct cytotoxicity was not performed for all patients due to the incidence of lymphopenia in ICU patients. However, we observed a very good correlation with degranulation assays, which may represent a good surrogate marker for cytotoxic function of NK cells through their degranulation capacities [45]. Second, we assessed NK immuno-monitoring in patients with severe sepsis and septic shock, but not in patients with non-severe sepsis who are usually not admitted to the ICU. These patients correspond to a less severe, but also to an earlier stage of sepsis, and might have presented the expected over-activated NK functional status as those observed in our non-septic SIRS patients. Thus, similar extensive functional studies, but done at an earlier times relative to onset of sepsis, or ideally, with serial timepoints, still need to be done. Third, partly due to severe lymphopenia, we did not assess functions of other cells (ie, monocytes, dendritic cells or Treg) that might have significant influence on NK cells functions. Finally, NK cells are present in the lungs at homeostasis, where their frequency is greater than in liver, peripheral blood mononuclear cells, spleen, or lymph nodes [9]. NK cells can be rapidly recruited to the sites of inflammation and we must keep in mind that, with regards to the concept of compartmentalization, that the status of NK cells within tissues may differ [10].

Overall, the present study provides the first report of extensive monitoring of the phenotype and functions of NK cells in critically-ill septic patients. Importantly, our results contrast with what has been reported in murine models [11]–[17]. Indeed, most murine models of septic shock have demonstrated a deleterious role of NK cells, with a protective effect on survival of NK-cell depletion. However, there are obvious differences between murine sepsis model and human data generated at bedside that could prevent direct comparison and/or explain apparent discrepancies. Conversely to patients that exhibit significant heterogeneity, mice are genetically identical, have same age and gender, are challenged in the same way (pathogen type, dose and route of administration) and present no confounding factors such as other treatments or comorbidities. Also, one of the major differences between the murine sepsis model and the human data provided here is the delay between the onset of sepsis and biological investigations. In the animal model, the timing is very short and controlled, whereas in patients, only the time from admission is known precisely whereas the time from sepsis onset can vary considerably. However, the timing of sampling in our study corresponded to “real-life” situations with regards to the development of future immuno-interventions. Transposed to human septic shock, the murine experiments might have prompted us to design an immuno-therapeutic trial with early NK depletion. Instead, the results of this work show that, in critically-ill septic patients, NK cells rapidly exhibit a normal or hypo-responsiveness status that may be part of the “immunoparalysis” (or tolerance), as reported for monocytes [6]–[8]. This hypo-responsiveness particularly involves patients with septic shock and IFN-γ secretion and is in complete agreement with the recent report of NK cell tolerance in term of inflammatory cytokines production (including IFN-γ) in a murine model of experimental bacterial sepsis [46]. These preliminary results and, aside from the supposed detrimental role of NK cells on the early amplification in the inflammatory response, the fact that NK cells also have beneficial anti-infectious (especially against CMV) as well as anti-inflammatory properties [47], actually support that therapeutic immuno-intervention in critically-ill septic patients could be directed towards stimulation of NK-cell functions [48].

## Supporting Information

Table S1 **Studies of NK cells in humans with Sepsis or SIRS.**
(DOCX)

Table S2 **Numbers of lymphocyte subpopulations at admission to the ICU in healthy, SIRS and Sepsis groups.**
(DOCX)

Table S3 **Serum cytokine levels in ICU patients with SIRS and Sepsis.**
(DOCX)

Methods S1 **Additional informations on immunological and statistical methodologies.**
(DOCX)
