# Supplementary material for: Phenotype and Functions of Natural Killer Cells in Critically-Ill Septic Patients
Source: PLoS One. 2012 Dec 6;7(12):e50446. doi: 10.1371/journal.pone.0050446 (PMC3516510; doi:10.1371/journal.pone.0050446)
Supplement: Table S1 — Studies of NK cells in humans with Sepsis or SIRS. (DOCX) [file pone.0050446.s001.docx]

| **Studies** | **Patients**  n / condition mean age severity | | | **Timing of sampling** | **Quantitative**  Absolute and/or % NK (definition) | **Qualitative**  Subset NK Cytotoxicity NK Cytokines  (Phenotype) (method) | | |
| --- | --- | --- | --- | --- | --- | --- | --- | --- |
| Klimpel^38^ 1986* | 23 Burns  20 controls | 36 | Burn surface 57% | 2/week | Normal %  (Leu 7 and 11+)‡ | NA | Decreased  (Cr51 4h against K562 and Raji HSV+) | NA |
| Bender^36^ 1988 | 15 Burns  17 controls | 37 | Burn surface 38% | Within first 2 days | Normal % | NA | Decreased  (Cr51 4h K562) | NA |
| Maturana^23^ 1991† | 11 Sepsis (SC, K n=2)  10 controls | 45 | Mortality 64% | NA | NA | NA | Decreased  (Cr51 4h K562) | NA |
| Holub^22^ 2003 | 32 Sepsis (S, no IDep)  34 controls | 33 | Apache II 16  SOFA 5  Mortality 6% | Day 1, 3, 7, 14 | Moderate NK lymphopenia, persistent at Day7  (CD3-CD56+CD16+) | NA | NA | NA |
| Takabayashi^37^ 2003 | 25 Surgery (K)  20 controls | 62 | Major Surgery | Before surgery and Day 1, 3, 7 | NA | NA | Decreased  (Cr51 4h K562) | NA |
| Giamerrelos^19^ 2006 | 49 Sepsis (SS)  6 controls | 77 | Apache II 20  SOFA 8 | Day 1 (12h) | Slightly increased %  (CD3-CD56+CD16+) | NA | NA | NA |
| Muller^25^ 2007 | 23 Sepsis (SC) | 64 | SOFA 10  Mortality 43% | Day 7, then 1/week | Increased %  CD56+CD16+? | NA | Decreased in 22/23  (Non-radioactive 3h K562) | NA |
| Venet^20^ 2010 | 21 Sepsis (SC)  11 controls | 58 | SAPSII 45  Mortality 19% | 2h after vasopressors then every 6h for Day 1 and 2 | Normal %  NK lymphopenia stable the 2 days (CD3-CD56+) | NA | NA | NA |
| Gogos^21^ 2010 | 505 Sepsis  (S:312 SS+SC:193, no IDep) | 65 | Apache II 16  Mortality 25% | Day 1 | NK lymphopenia in SS and SC  (CD3-CD56+CD16+) | NA | NA | NA |
| Andaluz^18^ 2011 | 50 Sepsis  (SS:12, SC:38) | 68 | Apache II 20  SOFA 8  Mortality 42% | Day 1, 3, 10 | Increased %  NK lymphopenia  (CD3-CD56+CD16+) | NA | NA | NA |
| De Pablo^39^ 2012 | 52 Sepsis (SC, no IDep)  36 controls | 62 | Apache II 25  SOFA 9  Mortality 34.6% | Day 1, 3, 7 | Normal %  NK lymphopenia  (CD3-CD56+) | CD57  CD69 | NA | NA |
| Current study | 29 Sepsis  (SS:14, SC:15, no IDep)  13 non-septic SIRS  21 healthy controls | 64 | SAPSII 45  SOFA 9  Mortality 21%  (For septic patients) | Within first 2 days | Normal %  NK lymphopenia  (CD3-CD56+CD16±) | Extensive phenotype ** | Normal for SS and SC †† (Degranulation CD107 assays, 4h, K562 and P815) ‡‡ | Decreased INFγ production, especially for SC †† |

*See also ref (34) (burns) or (35) (Trauma). †See also ref (24); ‡ Leu11 (CD16): nk and neutrophils, Leu7: NK and some T cells, Leu 19 (CD56); Cr51: chrome 51; K: cancer; NA: not available; No IDep: no underlying Immunodepression; S: Sepsis; SS: Severe sepsis; SC: Septic shock. ** See Table 2. ††Cytotoxicity (ADCC) and cytokinic (INF-γ) functions were increased in non-septic SIRS. ‡‡ Degranulation CD107 assay results were strongly correlated with CFSE-based non-radioactive tests (see Figure 1).
