# Supplementary material for: Phenotype and Functions of Natural Killer Cells in Critically-Ill Septic Patients
Source: PLoS One. 2012 Dec 6;7(12):e50446. doi: 10.1371/journal.pone.0050446 (PMC3516510; doi:10.1371/journal.pone.0050446)
Supplement: Table S2 — Numbers of lymphocyte subpopulations at admission to the ICU in healthy, SIRS and Sepsis groups. (DOCX) [file pone.0050446.s002.docx]

**Table S2:** Numbers of lymphocyte subpopulations at admission to the ICU in healthy, SIRS and Sepsis groups.

|  | Healthy  *n*=21 | SIRS group  *n*=13 | Sepsis group  *n*=29 | *p* |
| --- | --- | --- | --- | --- |
| Lymphocytes, mm^-3^ | 2100 [1882- 2528] | 985 [855- 1510]* | 830 [570- 1320] † | <0.001 |
| NK, % | 10.6 [6.9- 13.5] | 5.8 [4.5- 13.3] | 8.8 [4.0- 12.9] | ns |
| NK, mm^-3^ | 203 [149- 284] | 111 [57- 142]* | 69 [55- 106] † | <0.001 |
| CD2+, % | 85 [82- 86.7] | 85.2 [80.9- 86.5] | 85.1 [80.2- 90] | ns |
| CD2+, mm^-3^ | 1833 [1587- 2084] | 851 [692- 1273]* | 691 [487- 1188] † | <0.001 |
| CD3+, % | 75 [72-78] | 71 [67-80] | 75 [62-79] | ns |
| CD3+, mm^-3^ | 1332 [1098-1600] | 677 [538-1110]* | 594 [416-1016] † | <0.001 |
| CD3+DR+, % | 0.9 [0.5-1.1] | 0.8 [0.7-1.6] | 0.8 [0.5-1.4] | ns |
| CD3+CD4+, % | 44.8 [41.1-51.7] | 57.1 [50-60] | 54.5 [44.1-65.8] | ns |
| CD3+CD4+, mm^-3^ | 1069 [736-1233] | 558 [442-815]* | 519 [304-751] † | 0.001 |
| CD3+CD8+, % | 23.6 [21.3-28.7] | 10.8 [7.6-16.3]* | 15.1 [11.1-19.9] † | <0.001 |
| CD3+CD8+, mm^-3^ | 532 [411-653] | 120 [83-143]* | 151 [59-235] † | <0.001 |
| CD19+, % | 11.5 [9.3-14.5] | 9.9 [8.9-12.5] | 6.0 [4.0-9.1], † ‡ | 0.002 |
| CD19+, mm^-3^ | 241 [176-316] | 97 [79-161]* | 74 [30-97] † | <0.001 |
| DR+, % | 13.2 [10.7-14.8] | 9 [7.3-11.4]* | 8 [5-12.1] † | 0.012 |
| DR+, mm^-3^ | 285 [214-357] | 101 [67-156]* | 84 [57-139] † | <0.001 |
|  | | | | *p** |
| CD56+, % | - | 6.8 [6.5-15.7] | 14.7 [7.0-19.6] | ns |
| CD56+, mm^-3^ | - | 134 [72-208] | 116 [86-180] | ns |
| CD16+, % | - | 5.6 [5-13.4] | 9.6 [4.8-13.1] | ns |
| CD16+, mm^-3^ | - | 116 [57-141] | 80 [58-129] | ns |
| CD16+CD56+, % | - | 5.2 [4.4-11.7] | 8.6 [3.8-12.3] | ns |
| CD56+CD3+, % | - | 1.9 [0.8- 2.8] | 2.8 [1.4- 5.1] | ns |

Results are expressed as median [IQR] absolute number of cells or median [IQR] percentage of total lymphocyte count.

*p*: Comparison between healthy, SIRS and Sepsis groups by Kruskal-Wallis test.

*: p < 0.05, comparison between SIRS vs. healthy groups by Kruskal-Wallis post–hoc methods for multiple comparisons adjusted by step-up Simes method.

†: p < 0.05, comparison between Sepsis vs. healthy groups by Kruskal-Wallis post–hoc methods for multiple comparisons adjusted by step-up Simes method.

‡: p < 0.05, comparison between Sepsis vs. SIRS groups by Kruskal-Wallis post–hoc methods for multiple comparisons adjusted by step-up Simes method.

*p**: Comparison between Sepsis vs. SIRS groups by Mann-Whitney U test.
