# Supplementary material for: Phenotype and Functions of Natural Killer Cells in Critically-Ill Septic Patients
Source: PLoS One. 2012 Dec 6;7(12):e50446. doi: 10.1371/journal.pone.0050446 (PMC3516510; doi:10.1371/journal.pone.0050446)
Supplement: Table S3 — Serum cytokine levels in ICU patients with SIRS and Sepsis. (DOCX) [file pone.0050446.s003.docx]

**Table S3:** Serum cytokine levels in ICU patients with SIRS and Sepsis.

|  |  |  |  | Sepsis group (*n*=29) | |  |
| --- | --- | --- | --- | --- | --- | --- |
|  | SIRS group  *n*=13 | Sepsis group  *n*=29 | *p* | Severe sepsis  *n*=15 | Septic shock  *n*=14 | *p** |
| IL-1β | 0.2 [0.1-0.5] | 0.8 [0.4-1.1] | 0.003 | 0.7 [0.3-1] | 0.6 [0.2-1.1] | 0.012 |
| TNF-α | 1.5 [1-2.2] | 2.1 [1.5-2.9] | ns | 1.9 [1.6-2.2] | 2.6 [1.3-3.7] | ns |
| IL-6 | 41 [26-111] | 55 [26-215] | ns | 42 [25-85] | 202 [26-672] | 0.076 |
| IL-15 | 3.7 [2.4-4.2] | 3.3 [2.9-5.7] | ns | 3.2 [2.9-5.6] | 3.5 [2.9-6.9] | ns |
| IL-10 | 7.1 [3.3-9.7] | 8.7 [5.8-20.8] | ns | 10.1 [5.6-22.8] | 8.6 [6.6-11.9] | ns |
| TGF-β1 | 16625  [13168-28832] | 23617  [14188-34068] | ns | 24437  [13533-49527] | 20542  [14844-25264] | ns |
| IL-12 | 0.2 [0.17-0.25] | 0.3 [0.15-0.4] | ns | 0.33 [0.24-0.56] | 0.15 [0.1-0.22] | 0.035 |
| IL-18 | 308 [248-422] | 543 [302-633] | 0.057 | 553 [527-672] | 427 [209-565] | 0.054 |
| INF-γ | 0.1 [0.1-0.1] | 0.1 [0.1-0.1] | ns | 0.1 [0.1-0.1] | 0.1 [0.1-0.1] | ns |
| TGF-β2 | 129 [89-222] | 103 [74-167] | ns | 123.5 [77-204.9] | 85.7 [71.2-152.6] | ns |

Results are expressed as median [IQR] levels in pg/mL, except for INF-γ expressed in UI/mL.

*p*: Comparison between SIRS and Sepsis groups by Mann-Whitney U test. p*: Comparison between severe sepsis and septic shock groups by Mann-Whitney U test.
