# Supplementary material for: Phenotype and Functions of Natural Killer Cells in Critically-Ill Septic Patients
Source: PLoS One. 2012 Dec 6;7(12):e50446. doi: 10.1371/journal.pone.0050446 (PMC3516510; doi:10.1371/journal.pone.0050446)
Supplement: Methods S1 — Additional informations on immunological and statistical methodologies. (DOCX) [file pone.0050446.s004.docx]

**Methods**

*Study design*

The present study is a factorial study of the trial NTC00699868. 29 of the 42 ICU patients included in the present study were also evaluated longitudinally in a separate study [1].

*Definition of previous immunodeficiency*

An previous immunodeficiency was defined by the presence of at least one of the following conditions before ICU admission: pregnancy, neutropenia (white blood-cell counts <1 G/L or neutropenia <0.5 G/L), HIV seropositivity, use of immunosuppressive agents or corticosteroid therapy (if >0.5 mg/kg/d of prednisolone or equivalent for <1 month before admission, or any dosage if >1 month before admission), solid organ or bone-marrow transplantation, or solid cancer or hematologic disease with previous anticancer radiotherapy or chemotherapy.

*Definition and screening for nosocomial infections*

Bronchoalveolar lavage (BAL) was performed as soon as a patient presented with a suspicion of ventilator-associated pneumonia (VAP) and systematic screening for cytomegalovirus was done as previously reported [2]. Briefly, VAP was suspected if an association between a new pulmonary infiltrate or progression of an existing one on a chest radiograph and at least two of four criteria: fever >38°C or hypothermia <36°C, leukocytosis >10 G/L or <4 G/L, purulent secretions, and hypoxemia that required an increased positive end-expiratory pressure level and/or FiO_2_. The sampling area was selected based on the infiltrate location on the chest radiograph or the segment with purulent secretions visualized during bronchoscopy. BAL fluid was cultured quantitatively for bacteria, and diagnosis of bacterial VAP was established when a BAL quantitative culture grew at least one bacteria at a concentration of ≥10^4^ colony-forming units per mL. Blood was drawn for CMV and herpes simplex virus (HSV) serologies and for CMV antigenemia (pp65) in <48 h after ICU admission and then once a week (every Thursday) until discharge from the ICU. Active CMV infection was defined by a positive CMV pp65 antigenemia (at least one cell positive for CMV pp65 per 2x10^5^ leukocytes). When VAP was suspected, a shell-vial culture technique was used for BAL, with antibodies directed against immediate early antigens. Conventional CMV cultures were achieved with diploid human foreskin fibroblast cells monitored for up to 6 weeks for signs of infection. A BAL was considered positive for CMV when a cytopathogenic effect was seen on conventional cell cultures or when the shell-vial assay was positive.

*Assessment of sepsis and its severity*

At ICU admission, the following data were collected: age, gender, main reason for admission, severity of sepsis according to the ACCP-SCCM consensus [3], criteria for acute respiratory-distress syndrome (ARDS), use of mechanical ventilation or vasopressive agents, Simplified Acute Physiology Score (SAPS) II [4], and Sequential Organ-Failure Assessment score (SOFA) [5]. We also recorded ICU mortality, duration of mechanical ventilation, length of stay in the ICU, as well as occurrence of viral or bacterial nosocomial infections during the ICU stay. The diagnosis of sepsis and of its severity according to the usual definitions [3, 6], and of non-septic SIRS according to consensual criteria (ie, the presence of a compatible clinical setting and the absence of any microbiological documentation and antibiotherapy) was made by two independent investigators (FX and CG) who reviewed the charts.

*Definition of human NK cells subsets*

Frozen PBMC samples were thawed the day before analysis and incubated overnight in culture medium at 37°C. PBMC were then analyzed by 8-color-flow cytometry. We used a FACSCanto II cytometer (Becton Dickinson, Franklin Lakes, NJ, USA) with FacsDiva software (Becton Dickinson, Le Pont de Claix, France). The following monoclonal aantibodies were used: anti-CD56 (IgG1, B159), anti-CD3 (IgG1, SK7), anti-CD16 (IgG1, 3G8), anti-CD25 (IgG1, M-A251), anti-CD94 (IgG1, HP-3D9), anti-NKp46 (IgG1, 9E2), anti-NKG2D (IgG1, 1D11), from Becton Dickinson, San Diego, USA; anti-NKp30 (IgG1, AZ20), anti-NKp46 (IgG1, 9E2), anti-NKG2A (IgG2b, Z199), anti-NKG2D (IgG1, 1D11), anti-CD158a,h (IgG1, EB6), anti-CD158a (IgG1, 143211), anti-CD158e1e2 (IgG1, Z27.3.7), anti-CD158e (IgG1, DX9) from Beckman Coulter, Villepinte, France; and anti-NKG2C (IgG1, Fab 138C) from R&D systems, Abingdon, UK. The threshold for positivity was defined with species- and isotype-matched control mAbs for all stainings. Results were expressed as percentages of positive NK cells among all NK cells, or as mean fluorescence intensity (MFI) of certain markers.

*Assessment of NK cell effector function*

Natural cytotoxicity was assessed using MHC class I^-^human erythroleukemic K562 target cells (K562). Antibody-dependent cell cytotoxicity (ADCC) was assessed using P815 mouse mastocytoma cells (P815) coated with rabbit anti-mouse lymphocyte antibodies (Accurate Biochemicals, Westbury, NY). PBMCs were incubated for 4 h at 37°C in the presence of GolgiStop (1/1500; Becton Dickinson), anti-CD107 mAb (anti-CD107a, IgG1, H4A3; anti-CD107b, IgG1, H4B4, Becton Dickinson), and target cells (cell lines K562 and P815 were obtained from the American Type Culture Collection, Manassas, VA, USA). The effector–target ratio was 2.5:1. Cells were then washed in PBS supplemented with 2% FCS, 1 mM EDTA, and stained for 30 min at 4°C with PerCP-Cy5.5-conjugated anti-CD3 (Becton Dickinson), APC-conjugated anti-CD56 (Beckman coulter), and normal mouse serum 2% (Janvier, Le Genest-St-Isle, France). After fixation in 2% paraformaldehyde and permeabilization (PermWash; Becton Dickinson), the expression of IFN-γ in activated NK cells was detected by incubation with PE-conjugated anti-IFN-g (IgG1, 4S-B3, Becton Dickinson) for 30 min at 4°C. For both CD107 and INF-γ stains, results were expressed as percentages of positive cells within the whole NK-cell population.

*Assessment of NK cell cytotoxicity*

Briefly, target cell suspensions (K562 and P815) were labeled with 0.5 µM CFSE (Molecular Probes Europe, Leiden, the Netherlands) for 10 min at 37°C. After two washes, the CFSE-labeled target cells were re-suspended in assay medium in a round-bottomed well plate. PBMCs were added to yield effector–target (E:T) ratios of 50/1, 25/1,12.5/1, 6.25/1, and 3.125/1. Plates were incubated for 4 h in a humidified atmosphere of 5% CO_2_ at 37°C. Just before analysis, propidium iodide (2.5 μg/mL) was added to stain for dead cells. Samples were directly analyzed by flow cytometry. Controls, including only CFSE-stained target cells, were assayed to determine spontaneous cell death. Dead target cells were identified as being double stained with CFSE and PI, and specific cytotoxicity was calculated as the percentage of non-viable target cells after 4 h at the different E:T ratios minus spontaneous lysis (control).

*Assessment of serum level of cytokines*

TNF-α, interleukin 1 beta (IL-1β), interleukin 6 (IL-6), interleukin 10 (IL-10), interleukin 12 (IL-12), interleukin 15 (IL-15), interleukin 18 (IL-18), tumor-growth factors beta 1 and 2 (TGF β1 and TGF β2), and INF-γ were measured using commercial ELISA immunoassays (TNF, IL-1, IL-10, IL-12, IL-15 and TGF from R&D Systems, Abingdon, UK; IL-6 and INF-γ from Beckman Coulter, Immunotech, Marseille, France; IL-18 from MBL-Cliniscience, Montrouge, France). Assay limits of detection, as determined in the laboratory or following the manufacturers indications, were 0.5 pg/mL for TNF-α 0.1 pg/mL for IL-1β; 6 pg/mL for IL-6; 3 pg/mL for IL-10; 0.1 pg/ml for IL-12; 2 pg/mL for IL-15; 11 pg/ml for IL-18; 4.61 pg/mL for TGFβ1; 54 pg/mL for TGFβ2; and 0.1 IU/mL for INF-γ. For each assay below the limit of detection, the value of the limit of detection was used for statistical analysis.

*Statistical analyses*

We planned to include a minimum of 12–15 patients per group. This required enrolling 100 patients according to the usual incidences of septic shock, severe sepsis, and non-septic SIRS in our ICU and taking into account the other inclusion criteria (*i.e.*, absence of previous immunodeficiency and presence of CMV seropositivity on admission).

All statistical analyses were performed using SPSS 17.0 software (SPPS Inc., Chicago, IL, USA). For details of the SPSS Package provided by Marta Garcia-Granero, see <http://gjyp.nl/marta/>.

**References**

1- Chiche L, Forel JM, Thomas G, Farnarier C, Cognet C, et al.(2012) Interferon-γ Production by Natural Killer Cells and Cytomegalovirus in Critically Ill Patients. Crit Care Med (In press).

2- Chiche L, Forel JM, Roch A, Guervilly C, Pauly V, et al. (2009) Active cytomegalovirus infection is common in mechanically ventilated medical intensive care unit patients. Crit Care Med 37: 1850-1857.

3- Bone RC, Balk RA, Cerra FB, Dellinger RP, Fein AM, et al. (1992) Definitions for sepsis and organ failure and guidelines for the use of innovative therapies in sepsis. The ACCP/SCCM Consensus Conference Committee. American College of Chest Physicians/Society of Critical Care Medicine. Chest 101: 1644-1655.

4- Le Gall JR, Lemeshow S, Saulnier F. (1993) A new Simplified Acute Physiology Score (SAPS II) based on a European/North American multicenter study. JAMA 270: 2957-2963.

5- Vincent JL, Moreno R, Takala J, Willatts S, De Mendonça A, et al. (1996) The SOFA (Sepsis-related Organ Failure Assessment) score to describe organ dysfunction/failure. On behalf of the Working Group on Sepsis-Related Problems of the European Society of Intensive Care Medicine. Intensive Care Med 22: 707-710.

6- Levy MM, Fink MP, Marshall JC, Abraham E, Angus D, et al. (2003) SCCM/ESICM/ACCP/ATS/SIS. 2001 CCM/ESICM/ACCP/ATS/SIS International Sepsis Definitions Conference. Crit Care Med 31: 1250-1256.
